# Rat nasal mucosa‐derived ectodermal mesenchymal stem cells: A new therapeutic option for chronic rhinosinusitis

**DOI:** 10.1002/iid3.1337

**Published:** 2024-07-18

**Authors:** Liujin Li, ZhaoHui Liu, ChunLin Zhang, YiLin Long, TianWen Yang

**Affiliations:** ^1^ Affiliated Hospital of Zunyi Medical University Zunyi China

**Keywords:** cell‐based therapy, chronic rhinosinusitis, NM‐EMSCs

## Abstract

**Objective:**

To investigate the effect of nasal mucosa‐derived ectodermal mesenchymal stem cells (NM‐EMSCs) on the inflammatory state of rats with chronic rhinosinusitis (CRS) and the underlying therapeutic mechanism.

**Methods:**

NM‐EMSCs were isolated and extracted to construct a rat model of CRS. Fifteen Sprague‒Dawley (SD) rats were randomly divided into three groups: CK + NS group rats were injected locally with saline in the nasal mucosa; CRS + NS group rats were injected locally with saline in the nasal mucosa; and CRS + EMSCs group rats were injected locally with NM‐EMSCs in the nasal mucosa. One rat from the CRS + EMSCs group was randomly euthanized at 2, 4, and 6 days after injection, and the nasal mucosa tissues were collected for HE staining, Masson's trichrome staining, and periodic acid–Schiff staining.

**Results:**

NM‐EMSCs specifically expressing CD73, CD105, and CD90 were successfully isolated from the nasal mucosa of rats and were able to differentiate into adipocytes, osteoblasts, and chondrocytes. After saline and NM‐EMSC injection, compared with those in the blank control CK + NS group, the nasal mucosa in the CRS + NS and CRS + EMSC groups exhibited obvious thickening, a large amount of inflammatory cell infiltration, and increased collagen and mucin distribution. Four days post‐NM‐EMSC injection, the thickening of the nasal mucosa in the CRS group was gradually alleviated, the inflammatory cell infiltration gradually decreased, and the distribution of collagen and mucin and the collagen‐positive area gradually decreased. Moreover, only a small number of inflammatory cells were visible, and the distribution of mucins was limited to 6 days post‐NM‐EMSC injection.

**Conclusion:**

NM‐EMSCs effectively attenuated inflammation in the nasal mucosa of CRS model rats.

## Graphical abstract

We successfully obtained rat nasal mucosa‐derived ectodermal mesenchymal stem cells and found that they could ameliorate chronic sinusitis in rats.

## 1 INTRODUCTION

Chronic rhinosinusitis (CRS) is a common disease that is widely studied in the fields of otorhinolaryngology and head and neck surgery. CRS is a heterogeneous disease that is characterized by persistent inflammation of the nasal and paranasal sinus mucosa. The main manifestations are nasal congestion, runny nose, head and face pain, and decreased or lost sense of smell, and the course of the disease lasts for at least 12 weeks. The prevalence of CRS varies from country to country; it ranges from 8.0% to 10% in China,^1^, ^2^ with an overall prevalence of approximately 10.9% in Europe^3^ and 11.9% in the United States.^4^ CRS is an important global public health problem. Currently, CRS treatment includes drugs, surgery, and biological therapy, which have only been developed in recent years. Regardless of the treatment modality, inflammation cannot be effectively controlled in some patients, and this type of sinusitis is called refractory sinusitis. According to the European Positioning Paper on Rhinosinusitis and Nasal Paces (EPOS) 2012, more than 15% of CRS patients are refractory.^5^ Therefore, identifying more effective treatment modalities is crucial for the clinical improvement of CRS patients.

Ectomesenchymal stem cells (EMSCs) are mesenchymal stem cells derived from the ectoderm with multiple differentiation capabilities. EMSCs can be derived from the surface ectoderm and neural crest ectoderm.^6^ Neural crest ectoderm MSCs are distributed in the mucous membranes of the head and face, have multidirectional differentiation functions, and are capable of osteogenic and neuronal‐induced differentiation. Nasal mucosa‐derived ectodermal mesenchymal stem cells (NM‐EMSCs) are neural crest ectodermal mesenchymal stem cells that are easy to obtain, can proliferate quickly, and have high differentiation potential. Currently, domestic and international studies have focused on its multidirectional differentiation ability, and few studies on its immunomodulatory ability have been reported. Here, we explored the effect of NM‐EMSCs on CRS‐related inflammation through in vitro experiments to provide new ideas and methods for the treatment of CRS.

## 2 MATERIALS AND METHODS

### 2.1 Materials

Experimental animals: Fifteen healthy male Sprague‒Dawley (SD) rats (specific pathogen‐free grade, 6 weeks old, weighing approximately 200 g) were obtained from Yangzhou University Laboratory Animal Center. The rats were randomly divided into three groups of five rats each, and the groups were as follows: CK + NS group: healthy rats were injected locally with saline in the nasal mucosa; CRS + NS group: CRS model rats were injected locally with saline in the nasal mucosa; CRS + EMSCs group: CRS model rats were injected locally with NM‐EMSCs in the nasal mucosa.

### 2.2 Methodology

#### 2.2.1 Constructing the CRS model in SD rats

Anesthesia: The experimental animals were fasted for 12 h before the surgery, allowed to drink water freely, and subjected to respiratory anesthesia. After the rats (in the CRS group) were anaesthetized, the rats were immobilized on anatomical plates. *Staphylococcus aureus* was activated in Luria–Bertani (LB) medium, and after activation, it was cultured in LB medium for 16 h and then diluted in saline to a suspension of 1 MCF. Then, an *S. aureus*‐inoculated sponge was placed into the ostomeatal complex (OMC) of the left nasal cavity of the rats, and 0.5 mL of the bacterial suspension was slowly and carefully injected into the left nasal cavity with a 1 mL syringe. The control rats (CN group) were injected with an equal volume of saline via the same procedure. Blood was collected from the orbits for routine blood tests and Giemsa staining before surgery and 3 days, 4 weeks, 8 weeks, and 12 weeks after surgery.

#### 2.2.2 Isolation of NM‐EMSCs

Rat nasal mucosal tissues were obtained and placed in precooled PBS, after which the tissues were sheared. The erythrocytes were washed away, 0.25% trypsin was added, the mixture was digested at 37°C for 10 min, the digestion was terminated, and the mixture was centrifuged at 1200 rpm for 5 min. The supernatant was removed, and the mixture was resuspended in a complete medium, inoculated in culture flasks, and incubated routinely at 37°C in an incubator with 5% carbon dioxide. Afterward, the solution was changed every 2 days until the primary cells adhered to the bottom of the culture flask and reached 80% confluence, after which the cells were passaged.

#### 2.2.3 NM‐EMSC observation and detection

Stem cells were observed under a microscope at different times after primary isolation and after passage. The cells were passaged on slides and incubated for 24 h. The cells were fixed with 4% paraformaldehyde, permeabilized with 0.1% Triton X‐100/PBS for 15 min at room temperature, rinsed with PBS three times for 5 min, blocked with 5% BSA/PBS at 37°C for 30 min, diluted with 2% BSA/PBS at the appropriate ratio of CD73, CD105, and CD90 antibodies, and covered with coverslips overnight at 4°C in a wet box. The cells were then rinsed with PBS three times for 5 min each, rinsed with the corresponding fluorescein‐labeled secondary antibodies diluted at the appropriate ratio of 2% BSA/PBS, covered with coverslips, and incubated at 37°C for 1 h in a wet box. The cells were then rinsed with PBS three times for 5 min each. The cells were then subjected to Hoechst 33258 or DAPI staining, incubated at room temperature for 15 min, rinsed with PBS three times for 5 min, photographed under a microscope, and analyzed.

#### 2.2.4 Directed induction of NM‐EMSC differentiation

NM‐EMSCs were cultured continuously for 14−25 days in adipogenic, osteogenic, and chondrogenic induction media in vitro, and the medium was changed every 2 days. Then, oil red O staining, alizarin red staining, and toluidine blue staining were performed.

#### 2.2.5 NM‐EMSC injection

The intravenous injection of EMSCs was started after 12 weeks. Each rat was injected with 10^6^ cells; samples were taken for testing at 2, 4, and 6 days after injection.

#### 2.2.6 Tissue sampling and paraffin embedding

The tissue was removed and placed in 10% EDTA solution for decalcification. The decalcification solution was changed once every 3 days, and the decalcification time was approximately 28 days, which is when the tissue was easily penetrated through by a needle without any force. After decalcification, the tissue was sufficiently washed with PBS and then subjected to dehydration with an alcohol gradient series (75% for 1 h, 80% for 1 h, 90% for 1 h, 95% for 0.5 h, 100% for 20 min two times) and anhydrous ethanol + xylene (1:1 for 20 min). The tissue was sequentially cleared with xylene for 20 min and xylene for 60 min, followed by embedding in soft wax for 3 h and hard wax for 2 h. Then, the tissue was sequentially subjected to the following normal steps: cleaning, infiltration, and embedding. Finally, the paraffin‐embedded tissue was sectioned.

#### 2.2.7 HE staining, Masson's trichrome staining, periodic acid–Schiff (PAS) staining, and Giemsa staining

Briefly, after routine sections were deparaffinized and rehydrated, HE staining, Masson's trichrome staining, and PAS staining were performed according to the operating instructions of the kits (Solarbio). In addition, blood smears were prepared from the extracted blood; after natural drying, Giemsa staining was performed according to the instructions.

### 2.3 Statistical methods

GraphPad Prism 7.0 software was used for statistical analysis. The data are expressed as the mean ± standard deviation. One‐way ANOVA was used for comparisons between multiple groups, and the *t*‐test was used for comparisons between two groups, in which *p* < .05 indicated that the difference was statistically significant.

## 3 RESULTS

### 3.1 CRS modeling results

**Figure 1:**
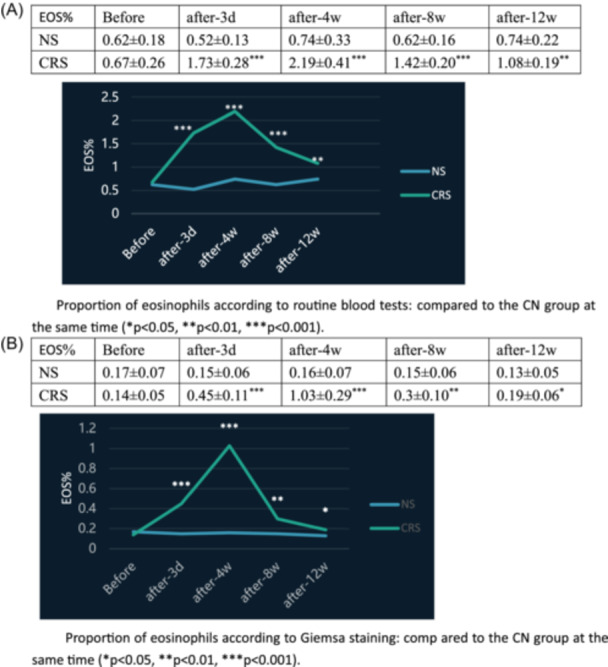
CRS rat modeling results: Routine blood tests and Giemsa staining results. (A) The proportion of eosinophils according to routine blood tests: Compared to the CN group at the same time (*p < .05, **p < .01, ***p < .001). (B) The proportion of eosinophils according to Giemsa staining: Compared to the CN group at the same time (*p < .05, **p < .01, ***p < .001). CRS, chronic rhinosinusitis.

**Figure 2:**
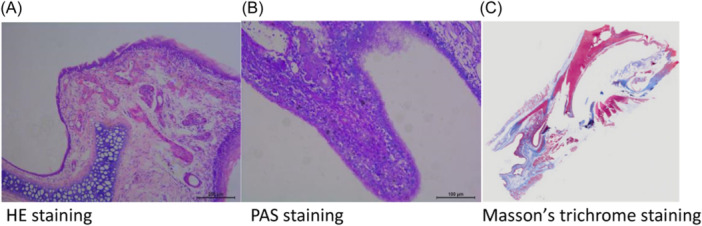
CRS rat modeling results: Pathological results of the nasal mucosa. (A) HE staining. (B) PAS staining. (C) Masson's trichrome staining. CRS, chronic rhinosinusitis; PAS, periodic acid–Schiff.

Blood was collected from the orbital area for routine blood tests and Giemsa staining before surgery and 3 days, 4 weeks, 8 weeks, and 12 weeks after surgery, and the results showed (Figure 1) that the proportion of eosinophils in the blood of the CRS group increased significantly at 3 days postinjection of *S. aureus* compared to that at 3 days postsaline injection; the proportion of eosinophils reached the highest level at 4 weeks and then began to decrease; nevertheless, the number of eosinophils was still greater than that in the control group at 12 weeks postsaline injection, and the difference was statistically significant (*p* < .05) compared with that of the CN group at the same time. The staining results revealed thickening of the nasal mucosa, with a large amount of inflammatory cell infiltration, collagen, and mucin in the CRS model rats at 12 weeks after *S. aureus* injection (Figure 2).

### 3.2 Isolation and identification of NM‐EMSCs

**Figure 3:**
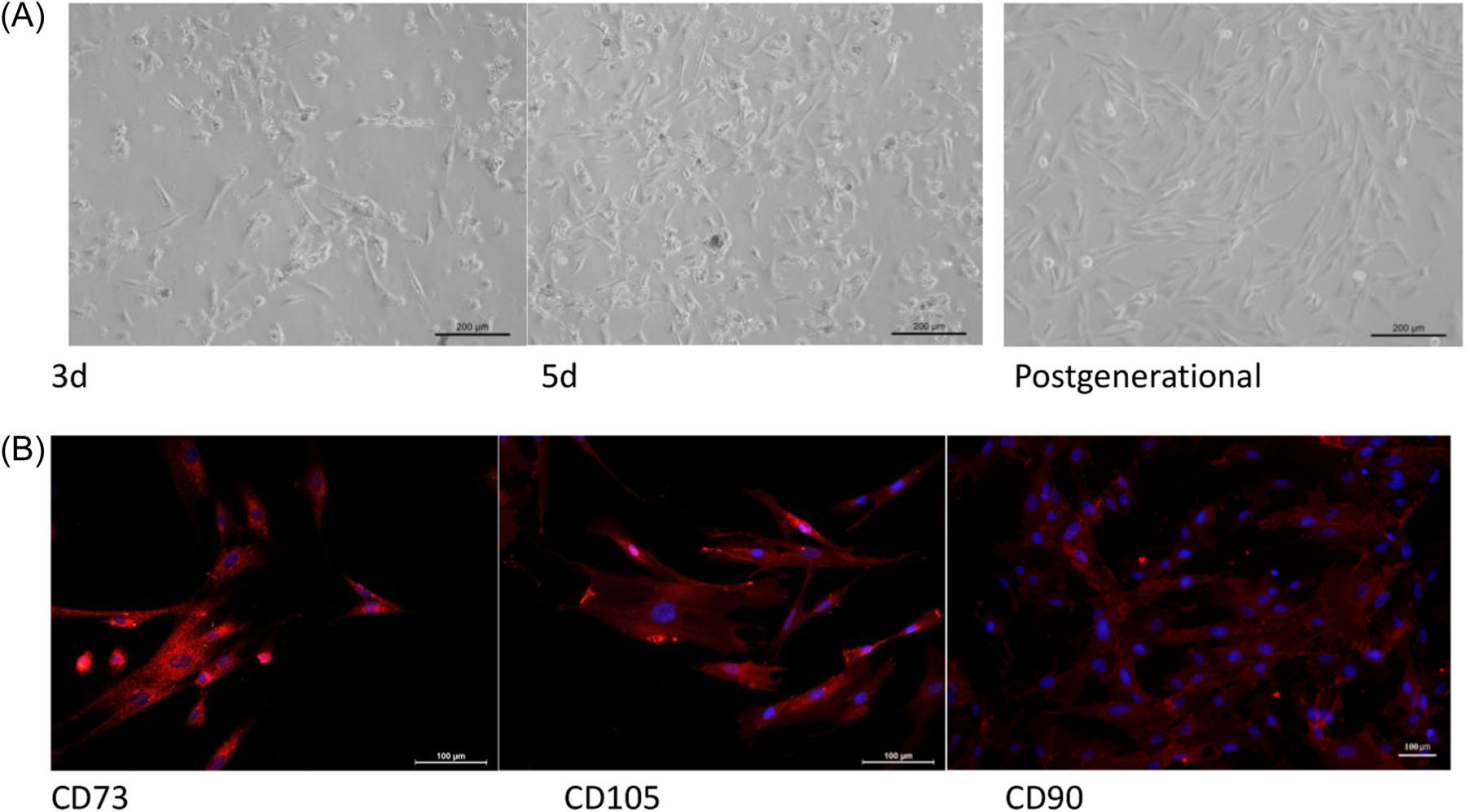
(A) Cell microscopy images were taken at ×100. (B) Fluorescence images were taken at ×200.

**Figure 4:**
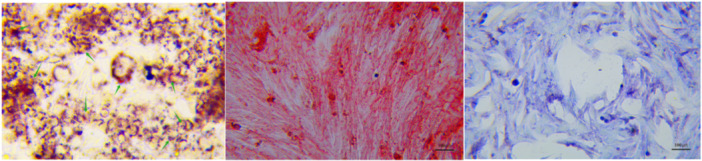
NM‐EMSCs cultured under adipogenic, osteogenic, and chondrogenic induction conditions were stained with oil red O (left panel, green arrows pointing to red lipid droplets), alizarin red (middle panel), or toluidine blue (right panel), respectively. NM‐EMSC, nasal mucosa‐derived ectodermal mesenchymal stem cells.

NM‐EMSCs were able to adhere to the bottom of the culture flask, and after incubation and passaging, the cells grew well and exhibited typical stem cell characteristics, and were spindle‐shaped (Figure 3A). After staining for CD73, CD105, and CD90 (Figure 3B), the cell type specificity of gene expression reached 99%, which indicated that the isolation and amplification of NM‐EMSCs were successful. NM‐EMSCs were successfully differentiated into adipocytes, osteoblasts, and chondrocytes when cultured under the indicated conditions (Figure 4).

### 3.3 Pathological findings

**Figure 5:**
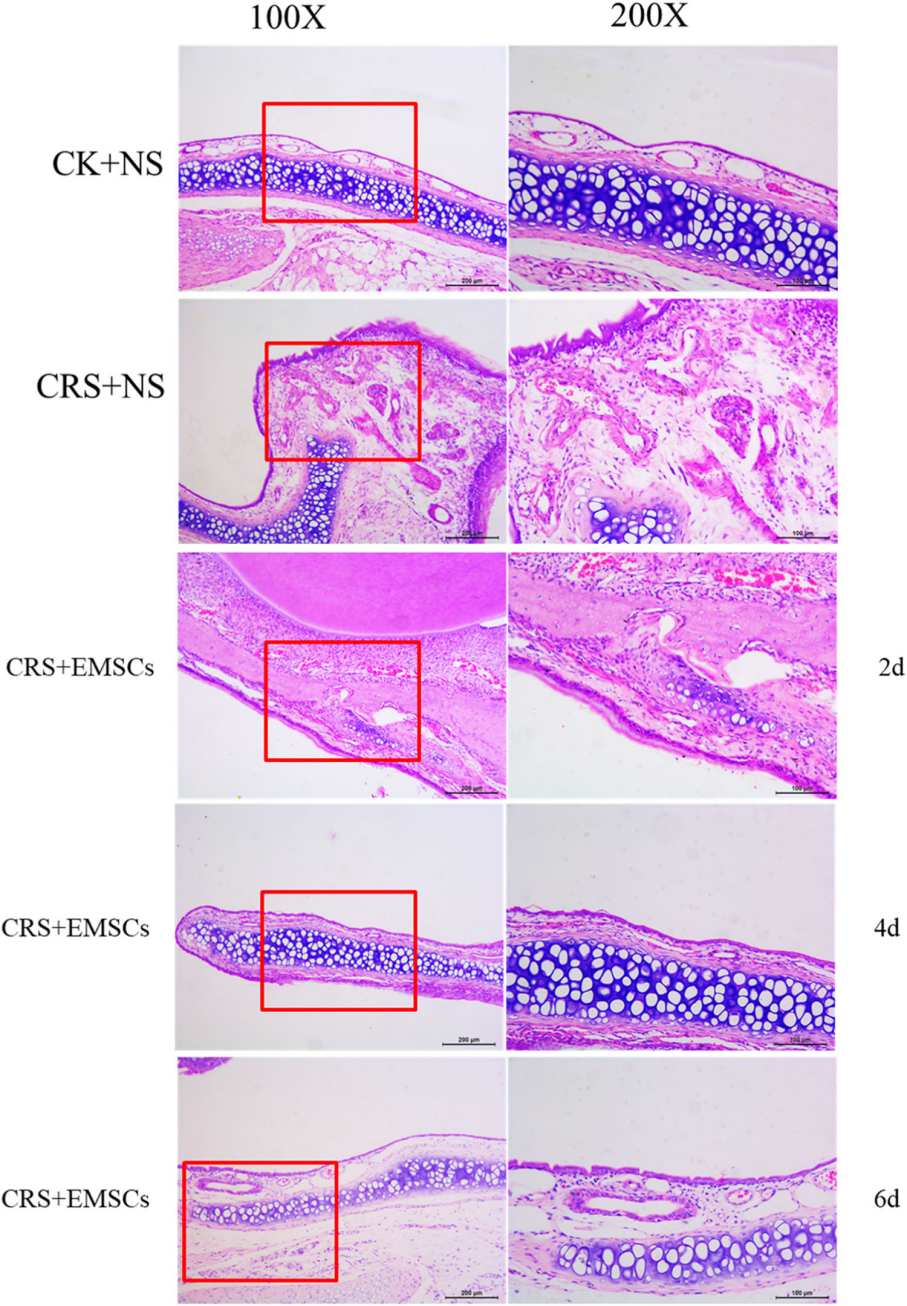
Effect of NM‐EMSC on nasal inflammation in CRS rats: HE staining results of rat nasal mucosa. CRS, chronic rhinosinusitis; NM‐EMSC, nasal mucosa‐derived ectodermal mesenchymal stem cells.

**Figure 6:**
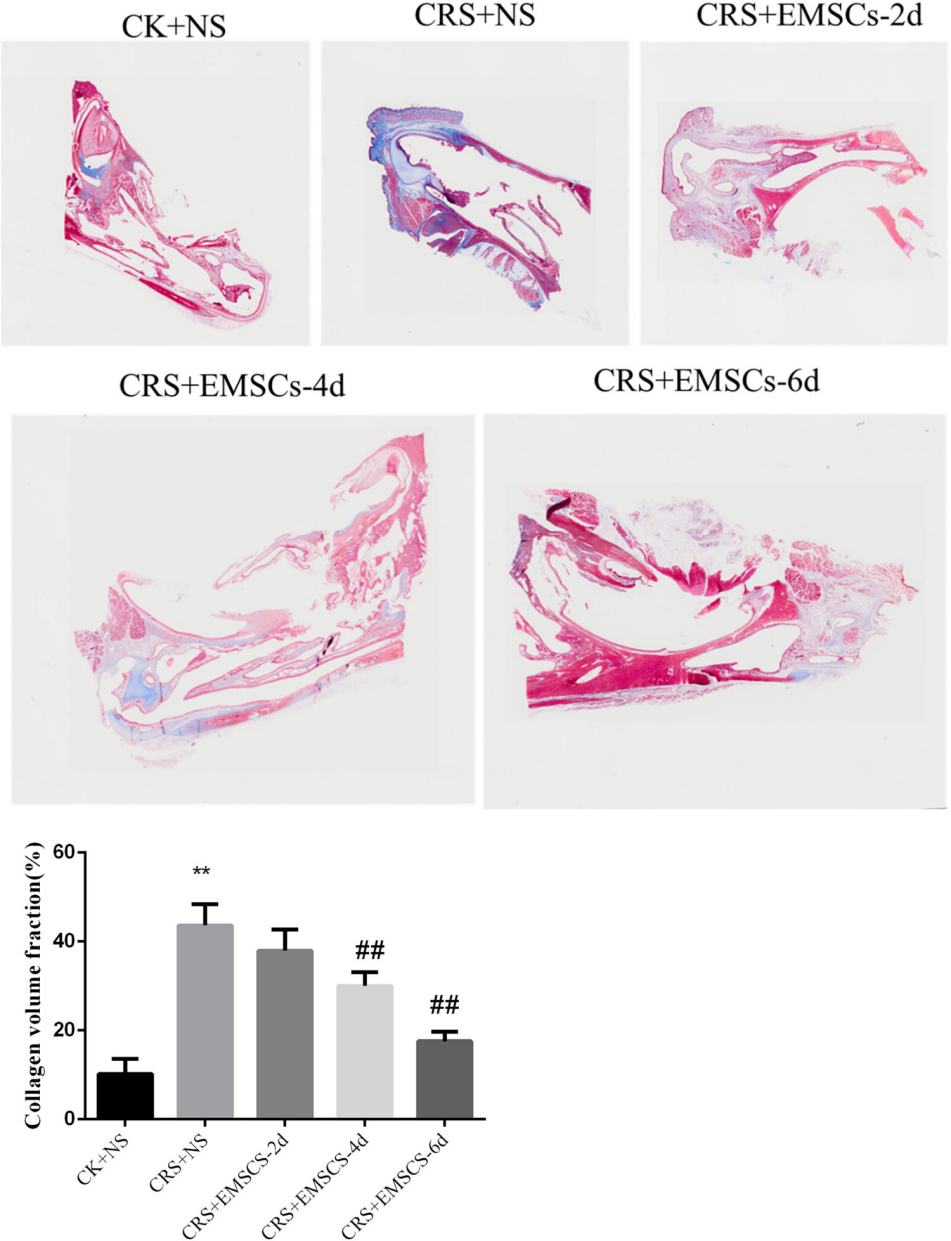
Effect of NM‐EMSC on nasal inflammation in CRS rats: Masson's trichrome staining results of rat nasal mucosa. **p < 0.01, compared to the CK+CN group. ^##^ *p* < 0.01, compared to the CRS+NS group. CRS, chronic rhinosinusitis; NM‐EMSC, nasal mucosa‐derived ectodermal mesenchymal stem cells.

**Figure 7:**
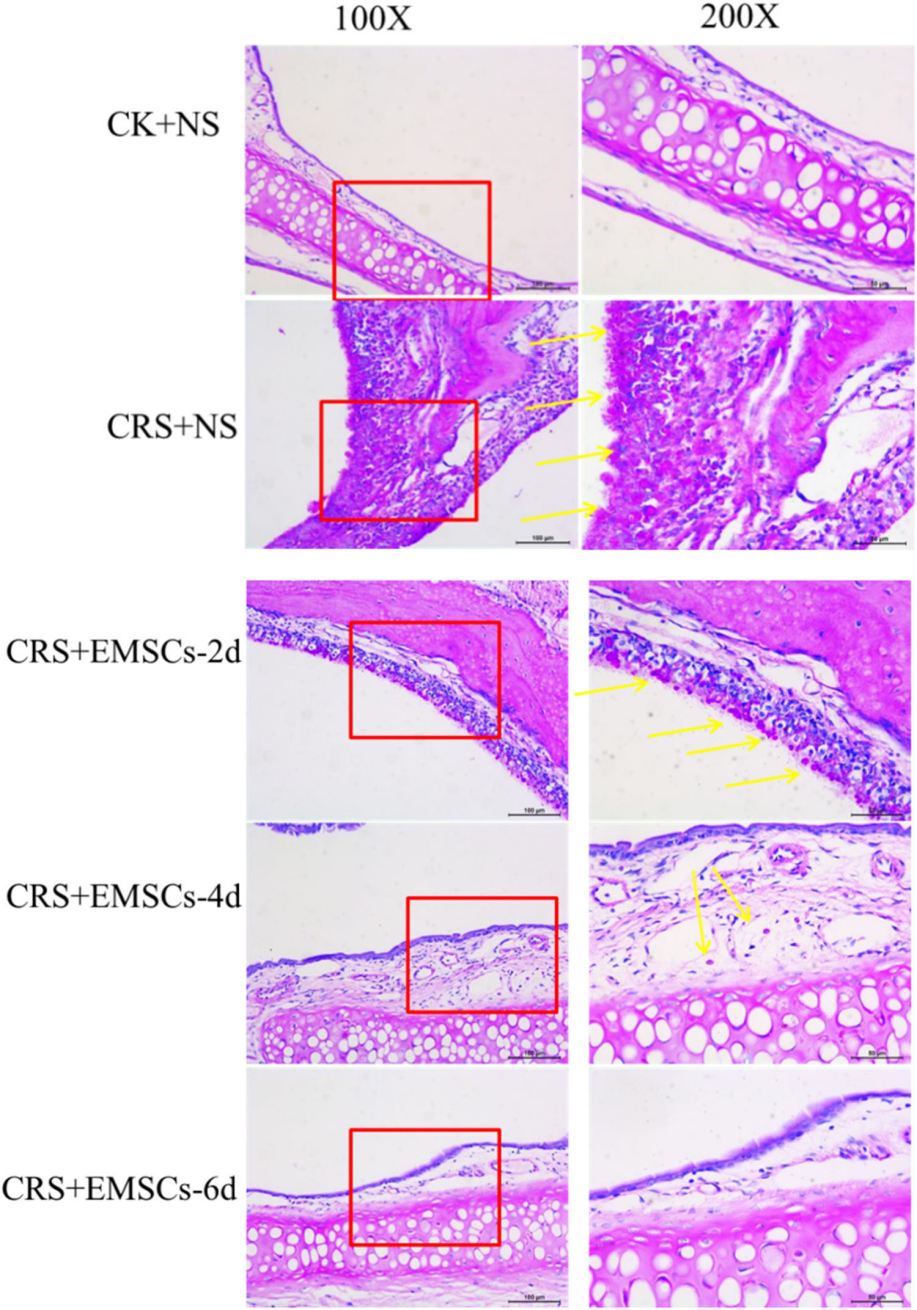
Effect of NM‐EMSC on nasal inflammation in CRS rats: PAS staining results of rat nasal mucosa. CRS, chronic rhinosinusitis; NM‐EMSC, nasal mucosa‐derived ectodermal mesenchymal stem cells; PAS, periodic acid–Schiff.

(1) HE staining revealed that (Figure 5) in the CK + NS group, the nasal mucosal tissue was basically intact, with a small amount of inflammatory cell infiltration; in the CRS + NS group, there was obvious thickening of the nasal mucosa, with a large amount of inflammatory cell infiltration, and mainly neutrophils and eosinophilic cells were observed. Two days after NM‐EMSC injection in the CRS + EMSC group, thickening of the nasal mucosa and inflammatory cell inflammation were still present, while there was a decrease in the number of eosinophilic cells. Thickening of the nasal mucosa was mitigated at 4 days after NM‐EMSC injection and inflammatory cell infiltration decreased. Thickening of the nasal mucosa was alleviated at 6 days post‐NM‐EMSC injection, and only a small number of inflammatory cells were visible.

(2) The Masson's trichrome staining results showed that (Figure 6) there was a small amount of collagen distributed in the nasal mucosa of the CK + NS group; there was obvious thickening of the nasal mucosa in the CRS + NS group, with collagen distributed around the thickened area of the nasal mucosa; and there was basically no difference in the collagen distribution in the nasal mucosa of the CRS + EMSCs group at 2 days after NM‐EMSC injection. At 4 days after NM‐EMSC injection, the thickening of the nasal mucosa was alleviated, and the collagen distribution was reduced; at 6 days after NM‐EMSC injection, the thickening of the nasal mucosa was alleviated, and the collagen distribution was further reduced. Analysis of the collagen‐positive area revealed that the difference was statistically significant (*p* < .05).
(3) PAS staining revealed (Figure 7) a small amount of mucin in the nasal mucosa of the CK + NS group and a large amount of mucin in the CRS + NS group. A large amount of mucin was observed in the nasal mucosa of the CRS + EMSCs group at 2 days post‐NM‐EMSC injection. However, the amount of mucin in the nasal mucosa was greatly reduced at 4 days after NM‐EMSC injection and only a small amount of mucin was visible in the nasal mucosa of the CRS + EMSCs group at 6 days after NM‐EMSC injection.

## 4 DISCUSSION

There are various methods for establishing sinusitis in animal models. In this study, we slightly changed the modeling method based on Chenjie et al.^7^ We inoculated *S. aureus* in the OMC of rats to establish a CRS model. The staining results revealed thickening of the nasal mucosa, with a large amount of neutrophil and eosinophilic cell infiltration, collagen, and mucin in CRS model rats at 12 weeks after *S. aureus* injection, which suggested that CRS was successfully induced in this study. Interestingly, during the modeling process, eosinophils in leukocytes tended to increase 4 weeks after *S. aureus* injection, and despite a clear tendency for them to decrease after 4 weeks, they were still more abundant than those in the control group at 12 weeks after *S. aureus* injection, and HE staining suggested that there was significant eosinophilic infiltration. This finding suggested that eosinophilic CRS (eCRS) was successfully induced in this study. Recent studies have shown^8^ that inflammation in CRS patients is highly heterogeneous and can be divided into three main inflammatory endotypes: the T1 endotype shows elevated Th1 cytokine levels; the T2 endotype shows eosinophilia and elevated Th2 cytokine levels; and the T3 endotype shows neutrophilia and elevated Th17 cytokine levels. eCRS is associated with severe eosinophil infiltration and refractoriness,^9^ and this group of patients also tends to exhibit a higher rate of recurrence, a phenomenon that is more common in European and American populations. Therefore, the animal model created in this study is representative of such patients.

The pathogenesis of CRS is complex due to many factors and is currently believed to be caused by mucosal barrier disruption due to interactions between different environmental factors and the host immune system, followed by chronic inflammation of the mucosa, which in most cases further leads to tissue remodeling and clinical symptoms.^10^ Among others, mucosal barrier disruption may be associated with allergens, microbial or viral infections, cytokines, hypoxia, or zinc deficiency.^11^ Mucosal remodeling may manifest as basement membrane thickening, fibrosis, and squamous metaplasia.^12^ Reducing mucosal remodeling, controlling mucosal inflammation, and repairing the mucosal barrier are essential for the alleviation of CRS, and the use of MSCs with immunomodulatory properties may be a new therapeutic option.

MSCs are high‐quality stem cells capable of continuous self‐renewal, multidirectional differentiation (osteoblastic, adipogenic, chondrogenic, and neuronal differentiation), and genetic stability^13^ and are ideal materials for regenerative medicine, in addition to their excellent immunomodulatory ability. Based on the results of numerous basic science studies, cell therapy with MSCs has also been successively tested in clinical trials; as of September 2023, 466 relevant clinical studies have been completed worldwide, and 220 studies are recruiting participants (www.clinicaltrials.gov). The results of a meta‐analysis that included 36 clinical studies, eight of which were randomized controlled trials (RCTs), showed that systemic infusion of MSCs is usually safe and that there was no association between MSC therapy and nonfebrile acute infusion toxicity, infections, or the development of malignancy.^14^ Moreover, the meta‐analysis was conducted again 8 years later with the inclusion of additional RCTs, and the results again showed that arteriovenous administration of MSCs is safe.^15^

MSCs can be derived from the ectoderm, mesoderm, and endoderm, and NM‐EMSCs are a type of ectodermal MSC. They have the advantages of easy accessibility, no or few ethical issues, stable genetic material, and low tumorigenicity.^16^, ^17^ NM‐EMSCs can be obtained from the mucosa of nasal turbinates, nasal cavities, sinuses, and other regions. Currently, domestic and international research on NM‐EMSCs has focused mainly on their differentiation characteristics and therapeutic effects on other diseases. Shi et al. reported that TG2 gene overexpression promoted the neural differentiation of NM‐EMSCs and facilitated the functional recovery of the spinal cord after spinal cord injury in rats.^18^ In addition, cyclic heat stress has been shown to activate the YAP signaling pathway to promote the differentiation of NM‐EMSCs into osteoblasts.^19^ Moreover, Xiang et al. reported that NM‐EMSCs exhibit phenotypes similar to those of other MSCs, as well as self‐renewal and multispectral differentiation abilities, and can secrete paracrine factors to promote hepatocyte regeneration, directional migration, and transdifferentiation into hepatocyte‐like cells in response to the liver microenvironment, thereby repairing or replacing damaged hepatocytes.^20^ NM‐EMSCs are derived from the neural crest ectoderm, and their neuronal differentiation has the natural advantage of not requiring transgenerational transformation; therefore, their role in nerve injury repair is a hot topic in current research. In addition, some scholars have shown that NM‐EMSCs have immunomodulatory effects. Yang et al. reported that nasal mucosa‐derived EMSCs can upregulate IgG‐2a and IFN‐γ and downregulate IgE, IgG 1, IL‐4, IL‐5, and IL‐10 to balance Th1 and Th2 immune responses in allergic rhinitis.^21^ Rui et al. reported that NM‐EMSCs can treat autoimmune arthritis in mice by increasing the percentage of Treg cells and decreasing TH1 and TH17 responses.^22^ Additionally, Veronica‐Elena Trombitaș first applied adipose‐derived stem cells (ADSCs) to a mouse model of CRS and demonstrated that ADSCs ameliorate inflammation in CRS model mice.^23^ However, there are no relevant studies on the effects of NM‐EMSCs on CRS‐related inflammation. We believe that NM‐EMSCs have a similar advantage in the treatment of CRS, so we carried out a study on NM‐EMSC intervention in CRS model rats and successfully isolated spindle‐shaped NM‐EMSCs specifically expressing CD73, CD105, and CD90 from the nasal mucosa of rats, which is in line with the results of Alvites et al.^13^ NM‐EMSCS was injected intravenously into the rat medial canthus after successful CRS modeling, and significant inhibition of mucosal remodeling and inflammation was observed in CRS model rats beginning on the fourth day after injection, as evidenced by the alleviation of nasal mucosal thickening and reductions in inflammatory cell infiltration, the collagen‐positive area, and the secretion of mucin. The above results suggest that NM‐EMSCs have an inhibitory effect on mucosal inflammation in CRS model rats and can effectively shorten the inflammatory cycle and accelerate the regression of CRS. However, the immunoregulatory mechanism of NM‐EMSCs is still unclear and deserves further in‐depth study to provide a theoretical basis for clinical trials.

Our study has several limitations that can be addressed in subsequent studies. First, there is a lack of validation of NM‐EMSC homing. Many studies have shown that when the body is ischemic, hypoxic, or injured, intra‐ or exogenous MSCs are predominantly distributed to the site of injury. Homing is crucial for the safe and effective use of MSCs in the clinic. Second, CRS is a highly heterogeneous disease, and the CRS model in this study does not completely represent all types of CRS. The efficacy of NM‐EMSCS in treating other types of CRS needs to be further explored to clarify the overall efficacy of NM‐EMSCS in treating CRS.

## 5 CONCLUSIONS

CRS is a common disease in the field of otorhinolaryngology and head and neck surgery, and some patients are difficult to benefit from existing treatments. Our study demonstrated that rat NM‐EMSCs were effective in alleviating nasal mucosal inflammation, shortening the inflammatory cycle, and favoring the clinical regression of CRS in CRS model rats. NM‐EMSCS is a promising stem cell type that is a noninvasive and readily accessible cell population, unlike other sources of stem cells such as bone marrow or adipose tissue. This unique characteristic promotes its potential clinical application and minimizes patient discomfort and trauma during cell harvesting. NM‐EMSCs are good materials for stem cell biotherapy applied to CRS. The results of this animal experiment provide a research basis for the next step of clinical research and a new direction for new options in the clinical treatment of CRS.

## AUTHOR CONTRIBUTIONS

**Liujin Li**: Conceptualization; methodology; project administration; writing—original draft; writing—review and editing. **ZhaoHui Liu**: Conceptualization; project administration; writing—review and editing. **ChunLin Zhang**: Methodology; validation. **YiLin Long**: Formal analysis; software. **TianWen Yang**: Data curation; formal analysis.

## ETHICS STATEMENT

The study was conducted according to the guidelines of the Declaration of Helsinki and approved by the Ethics Committee of the Zunyi Medical University (Appl. No.:ZMU21‐2112‐001).

## Data Availability

The authors confirm that the data supporting the findings of this study are available in the article.
